# Multidrug Resistance Plasmid pTZC1 Could Be Pooled among *Cutibacterium* Strains on the Skin Surface

**DOI:** 10.1128/spectrum.03628-22

**Published:** 2023-02-27

**Authors:** Juri Koizumi, Keisuke Nakase, Nobukazu Hayashi, Chikage Takeo, Hidemasa Nakaminami

**Affiliations:** a Department of Clinical Microbiology, School of Pharmacy, Tokyo University of Pharmacy and Life Sciences, Hachioji, Tokyo, Japan; b Department of Dermatology, Toranomon Hospital, Tokyo, Japan; c Yoyogiuehara Dermatology Clinic, Shibuya-ku, Tokyo, Japan; Tianjin University

**Keywords:** acne vulgaris, *Cutibacterium*, multidrug resistance, pTZC1, transconjugation

## Abstract

Acne vulgaris is a chronic inflammatory skin disease that is exacerbated by Cutibacterium acnes. Although antimicrobials such as macrolides, clindamycin, and tetracyclines are used to treat acne caused by *C. acnes*, the increasing prevalence of antimicrobial-resistant *C. acnes* strains has become a global concern. In this study, we investigated the mechanism by which interspecies transfer of multidrug-resistant genes can lead to antimicrobial resistance. Specifically, the transfer of pTZC1 between *C. acnes* and *C. granulosum* isolated from specimens of patients with acne was investigated. Among the *C. acnes* and *C. granulosum* isolated from 10 patients with acne vulgaris, 60.0% and 70.0% of the isolates showed resistance to macrolides and clindamycin, respectively. The multidrug resistance plasmid pTZC1, which codes for macrolide-clindamycin resistance gene *erm*(50) and tetracycline resistance gene *tet*(W), was identified in both *C. acnes* and *C. granulosum* isolated from the same patient. In addition, whole-genome sequencing revealed that the pTZC1 sequences of *C. acnes* and *C. granulosum* showed 100% identity using comparative whole-genome sequencing analysis. Therefore, we hypothesize that the horizontal transfer of pTZC1 between *C. acnes* and *C. granulosum* strains may occur on the skin surface. The plasmid transfer test revealed a bidirectional transfer of pTZC1 between *C. acnes* and *C. granulosum*, and transconjugants that obtained pTZC1 exhibited multidrug resistance. In conclusion, our results revealed that the multidrug resistance plasmid pTZC1 could be transferred between *C. acnes* and *C. granulosum*. Furthermore, since pTZC1 transfer among different species may aid in the prevalence of multidrug resistant strains, antimicrobial resistance genes may have been pooled on the skin surface.

**IMPORTANCE** The emergence of antimicrobial resistance not only in Cutibacterium acnes strain but also other skin bacteria such as Staphylococcus epidermidis is a big concern due to antimicrobial use for the treatment of acne vulgaris. Increased prevalence of macrolides-clindamycin resistant *C. acnes* relates to the acquisition of exogenous antimicrobial resistance genes. *erm*(50) is harbored by the multidrug resistance plasmid pTZC1, which has been found in *C. acnes* and *C. granulosum* strains isolated from patients with acne vulgaris. In this study, *C. acnes* and *C. granulosum* with pTZC1 were found in the same patient, and plasmid transfer between *C. acnes* and *C. granulosum* was proved by transconjugation assay. This study showed plasmid transfer between other species and the possibility of further prevalence antimicrobial resistance between *Cutibacterium* species.

## INTRODUCTION

Acne vulgaris is a chronic inflammatory skin disease that affects approximately 85% of adolescents (1). Multiple factors are involved in the development and exacerbation of acne vulgaris, including increased sebum secretion by androgens, hyperkeratinization, and bacterial overgrowth (2). Notably, Cutibacterium acnes, a skin inhabitant of hair follicles, is associated with exacerbated inflammation in acne vulgaris. For acne treatment, benzoyl peroxide and topical retinoid, including adapalene, are used to ameliorate hyperkeratinization, while antimicrobial agents target *C. acnes* in the acute inflammatory phase. Among the antimicrobial agents, topical clindamycin, nadifloxacin, and ozenoxacin, in addition to oral doxycycline, minocycline, and roxithromycin, are recommended by the Japanese acne treatment guidelines (3). However, the emergence and increase of antimicrobial-resistant *C. acnes* strains have become a great concern, and antimicrobial use for acne treatment is strongly related to these phenomena (4 to 7). *Cutibacterium* species, such as *C. avidum*, *C. granulosum*, *C. modestum*, and *C. namnetense*, in addition to *C. acnes*, are commensal skin bacteria (8, 9). *Cutibacterium* species other than *C. acnes* are rarely found in acne lesions.

Antimicrobial-resistant *C. avidum* and *C. granulosum* strains are more frequently found in patients with acne vulgaris than *C. acnes* (10). Furthermore, antimicrobial-resistant Staphylococcus epidermidis, a major skin commensal bacterium, was isolated more frequently from patients with acne vulgaris than from healthy individuals (11). Thus, the efficient use of antimicrobial agents for acne treatment is closely related to the development of antimicrobial resistance in various skin inhabitants, including *C. acnes*.

*C. acnes* can develop antimicrobial resistance through various mechanisms. Mutations in 23S rRNA and acquisition of ribosomal methylase coding genes *erm*(X) and *erm*(50) are well-known mechanisms of resistance against the macrolides-clindamycin (12, 13). *erm*(X) is found on chromosomes or linear plasmids and is transmitted between *C. acnes* strains (14, 15). In contrast, *erm*(50) and the tetracycline resistance gene *tet*(W) are only found in *C. acnes* and are located on pTZC1. pTZC1 also carries a type-IV secretion system operon, which is associated with conjugative transfer among *C. acnes* strains (16).

In this study, we showed the coexistence of *C. acnes* and *C. granulosum* in acne pustules and the transference of pTZC1 between different strains of *Cutibacterium* species.

## RESULTS

### Antimicrobial susceptibility of *Cutibacterium* strains isolated from patients with acne vulgaris.

The *C. acnes* and *C. granulosum* strains were isolated simultaneously from specimens isolated from the acne pustules of 10 patients with acne vulgaris, of which four were diagnosed with severe (40.0%) and three with moderate and mild (30.0%) acne (17). The antimicrobial susceptibility of these strains was determined (Table 1). Clarithromycin- and clindamycin-resistant strains were found in 60.0% (6/10) and 70.0% (7/10) of the *C. acnes* strains and 40.0% (4/10) and 50% (5/10) of the *C. granulosum* strains, respectively. The rates of resistance to clarithromycin and clindamycin in the *C. granulosum* strains were higher than those in the *C. acnes* strains. In addition, doxycycline-resistant strains have been identified in *C. acnes* and *C. granulosum* isolated from an one patient out of ten patients.

**TABLE 1:** Profiles of *C. acnes* and *C. granulosum* strains isolated from the same patients with acne vulgaris^*a*^

			MIC (μg/mL)			
Strains	Species	SLST	Clarithromycin	Clindamycin	Doxycycline	Minocycline
TP-CU426	*C. acnes*	F4	≥256	≥256	16	2
TP-CG7	*C. granulosum*	-	≥256	4	32	8
TP-CU428	*C. acnes*	A2	≥256	2	1	2
TP-CG8	*C. granulosum*	-	≤0.06	≤0.06	1	1
TP-CU476	*C. acnes*	A44	≥256	64	0.25	0.25
TP-CG11	*C. granulosum*	-	≥256	≥256	1	1
TP-CU504	*C. acnes*	A2	≥256	≥256	0.13	0.13
TP-CG12	*C. granulosum*	-	≥256	2	0.25	0.13
TP-CU814	*C. acnes*	F1	≥256	128	0.5	0.5
TP-CG108	*C. granulosum*	-	≥256	≥256	1	2
TP-CU754	*C. acnes*	H1	≥256	32	0.5	0.25
TP-CG105	*C. granulosum*	-	64	128	0.5	0.5
TP-CU755	*C. acnes*	A2	≤0.06	≤0.06	0.25	0.13
TP-CG106	*C. granulosum*	-	64	128	0.5	0.5
TP-CU762	*C. acnes*	A5	≤0.06	≤0.06	0.25	0.13
TP-CG107	*C. granulosum*	-	≤0.06	≤0.06	≤0.06	≤0.06
TP-CU823	*C. acnes*	A5	≤0.06	0.25	0.5	0.25
TP-CG109	*C. granulosum*	-	≤0.06	≤0.06	≤0.06	≤0.06
TP-CU913	*C. acnes*	F4	≤0.06	≤0.06	0.25	≤0.06
TP-CG113	*C. granulosum*	-	≥256	8	≤0.06	≤0.06

a -, not applicable.

### Antimicrobial resistance factors in *Cutibacterium* strains.

The resistance factors of macrolides-clindamycin and tetracyclines were analyzed (Table 2). While 23S rRNA mutations were found in four and two *C. acnes* and *C. granulosum* isolates, respectively, *erm*(X) was detected in three *C. granulosum* strains. Moreover, the pTZC1 harboring *erm*(50) and *tet*(W) was found in both C. acnes and C. granulosum strains isolated from the specimens isolated from two patients.

**TABLE 2:** Identification of macrolides-clindamycin resistance factors and pTZC1^*a*^

Strains	Species	Resistance factor and pTZC1				
		23S rRNA mutation	*erm*(X)	*erm*(50)	*tet*(W)	*traE*
TP-CU426	*C. acnes*	ND	−	+	+	+
TP-CG7	*C. granulosum*	A2058T	−	+	+	+
TP-CU428	*C. acnes*	A2058G	−	−	−	−
TP-CG8	*C. granulosum*	ND	−	−	−	−
TP-CU476	*C. acnes*	A2058G	−	−	−	−
TP-CG11	*C. granulosum*	ND	+	−	−	−
TP-CU504	*C. acnes*	A2058G	−	−	−	−
TP-CG12	*C. granulosum*	A2058T	−	−	−	−
TP-CU814	*C. acnes*	ND	−	+	+	+
TP-CG108	*C. granulosum*	ND	+	+	+	+
TP-CU754	*C. acnes*	A2058T	−	−	−	−
TP-CG105	*C. granulosum*	ND	−	−	−	−
TP-CU755	*C. acnes*	ND	−	−	−	−
TP-CG106	*C. granulosum*	ND	+	−	−	−
TP-CU913	*C. acnes*	ND	−	−	−	−
TP-CG113	*C. granulosum*	A2058G	−	−	−	−

a Acquisition of pTZC1 was confirmed by detection of three genes corded (*erm*(50), *tet*(W), and *traE*). ND, not detected.

### Whole-genome sequence analysis.

**FIG 1:**
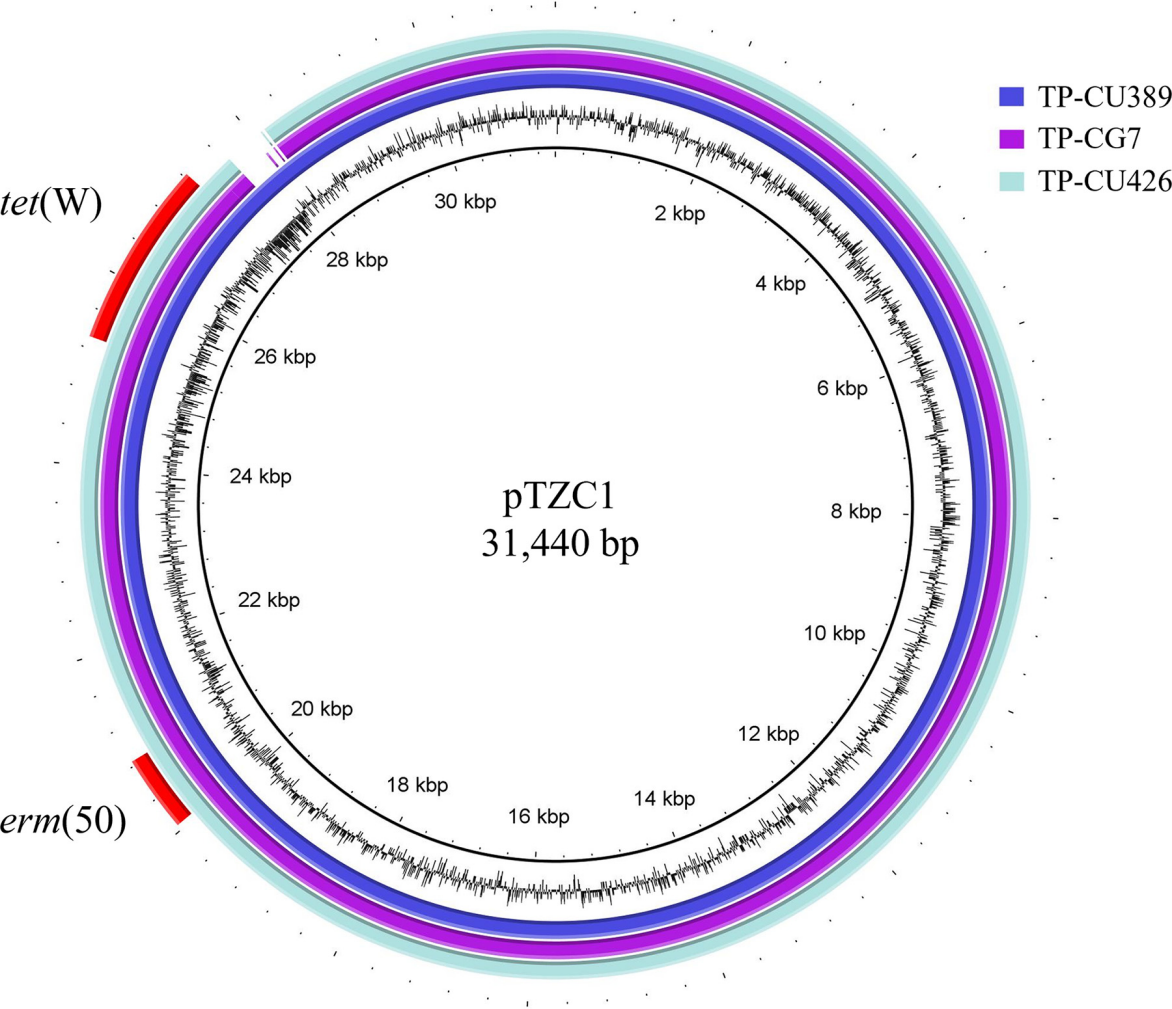
Comparison of pTZC1 sequences. pTZC1 from *C. acnes* TP-CU426 and *C. granulosum* TP-CG7 isolated from the same patient were compared to pTZC1 isolated from *C. acnes* TP-CU389. The sequences from TP-CU426 and TP-CG7 had deletion with approximately 500 bp compared with that of TP-CU389.

Whole-genome sequencing revealed that both *C. acnes* TP-CU426 and *C. granulosum* TP-CG7 had a chromosome (2,495,124 bp, with a GC content of 60.0%; and 2,148,765 bp, with a GC content of 64.1%, respectively) and a plasmid (30,947 bp, with a GC content of 65.0%). The coverage levels were 1,403 and 1,633 for *C. acnes* TP-CU426 and *C. granulosum* TP-CG7, respectively. The genome sequences of the chromosomes and pTZC1 from *C. acnes* TP-CU426 and *C. granulosum* TP-CG7 were deposited in NCBI GenBank under accession numbers AP026712, AP026713, AP026710, and AP026711. The pTZC1 sequence of *C. acnes* TP-CU426 (accession no. AP026713) and *C. granulosum* TP-CG7 (accession no. AP026711) showed 100% identity (30,947/30,947 bp) (Fig. 1 and supplemental material). In contrast to the pTZC1 from the *C. acnes* TP-CU389 (accession no. LC473083), these sequences contained a 500-bp deletion. This region was located on the 16,115 to 16,668 bp region of pTZC1 from the *C. acnes* TP-CU389 and was estimated to be a noncoding sequence using BLAST analysis.

Resistance factors conferring doxycycline resistance were analyzed in these strains (18). The *C. acnes* TP-CU426 and *C. granulosum* TP-CG7 had mutations in *rpsJ* (A170T, K57M; T172G, Y58D) but not in 16S rRNA. The upstream sequences of *tet*(W) are known to contribute to tetracycline resistance (4). When upstream sequences of *tet*(W) were compared, those sequences in the *C. acnes* TP-CU426 and *C. granulosum* TP-CG7 were identical to those of group Biii reported by Aoki et al. (4).

### Transconjugation of *erm*(50) between *C. acnes* and *C. granulosum* strains.

pTZC1 found in the two species from the same patient had identical sequences with the same 500 bp deletion. This provided strong evidence that the plasmid was transferred between *C. acnes* and *C. granulosum* strains on the skin surface. Thus, the transferability of pTZC1 between *C. acnes* and *C. granulosum* strains was examined (Table 3).

**TABLE 3:** Transconjugation frequency between *C. acnes* and *C. granulosum* clinical isolates^*a*^

Strains	Transconjugation frequency	MIC (μg/mL)			
		Clarithromycin	Clindamycin	Doxycycline	Minocycline
*C. acnes* TP-CU389 (D)		≥256	≥256	0.5	0.13
*C. acnes* ATCC11828 (R)		≤0.06	≤0.06	0.13	≤0.06
*C. acnes* transconjugant	1.2 × 10^−5^ ± 7.8 × 10^−6^	≥256	≥256	0.5	0.13
*C. granulosum* ATCC25564 (R)		≤0.06	≤0.06	0.25	≤0.06
*C. granulosum* transconjugant	4.8 × 10^−6^ ± 6.5 × 10^−6^	≥256	≥256	1	1
*C. granulosum* TP-CG10 (D)		≥256	≥256	32	8
*C. acnes* ATCC11828 (R)		≤0.06	≤0.06	0.13	≤0.06
*C. acnes* transconjugant	2.7 × 10^−5^ ± 4.7 × 10^−6^	≥256	≥256	16	8
*C. granulosum* ATCC25564 (R)		≤0.06	≤0.06	0.25	≤0.06
*C. granulosum* transconjugant	5.9 × 10^−5^ ± 3.7 × 10^−6^^*b*^	≥256	≥256	1	0.5
*C. granulosum* TP-CG7 (D)		≥256	4	32	8
*C. acnes* ATCC11828 (R)		≤0.06	≤0.06	0.13	≤0.06
*C. acnes* transconjugant	2.1 × 10^−5^ ± 2.8 × 10^−5^	≥256	≥256	16	2
*C. acnes* TP-CU426 (D)		≥256	≥256	16	2
*C. granulosum* ATCC25564 (R)		≤0.06	≤0.06	0.25	≤0.06
*C. granulosum* transconjugant	4.0 × 10^−6^ ± 3.7 × 10^−6^	≥256	≥256	8	4
*C. granulosum* TP-CG108 (D)		128	≥256	1	2
*C. acnes* ATCC11828 (R)		≤0.06	≤0.06	0.13	≤0.06
*C. acnes* transconjugant	6.8 × 10^−5^ ± 4.2 × 10^−5^^*b*^	≥256	≥256	0.5	0.25
*C. acnes* TP-CU814 (D)		≥256	128	0.5	0.5
*C. granulosum* ATCC25564 (R)		≤0.06	≤0.06	0.25	≤0.06
*C. granulosum* transconjugant	3.6 × 10^−4^ ± 1.6 × 10^−4^	≥256	128	16	8

a D, donor; R, recipient.

b Significantly difference versus *C. acnes* TP-CU389 to *C. acnes* ATCC11828 using Welch’s *t* test (*P* < 0.05).

Thus, we confirmed that pTZC1 could be transferred in both directions between *C. acnes* and *C. granulosum in vitro*. Furthermore, the pTZC1 was transferred between *C. acnes* and *C. granulosum* strains. Transconjugants were obtained from all the donors. The frequency of transconjugation differed according to the recipient strain. All the transconjugants showed high MIC values for clarithromycin and clindamycin. In contrast, the MIC values for doxycycline and minocycline in transconjugants were higher in the donor strains than in recipient strains and varied with the strain. These results strongly suggested that pTZC1 could be transferred between the *C. acnes* and *C. granulosum* strains on the skin and confer resistance to macrolides and clindamycin.

### Growth ability of transconjugants and expression of pTZC1.

The effect of pTZC1 acquisition was analyzed by calculating the doubling time and copy number of pTZC1 (Table 4). Differences in the doubling times were within 2-fold for all strains, indicating that pTZC1 acquisition did not affect the growth ability.

**TABLE 4:** The doubling time and copy number of pTZC1 in strains used for transconjugation experiments^*a*^

Strains	Doubling time	Copy no. of pTZC1
*C. acnes* TP-CU389 (D)	3.8 ± 0.3	0.79 ± 0.31
*C. acnes* ATCC11828 (R)	6.0 ± 2.0	
*C. acnes* transconjugant	6.1 ± 0.7	0.69 ± 0.18
*C. granulosum* ATCC25564 (R)	4.3 ± 1.3	
*C. granulosum* transconjugant	4.9 ± 0.7	1.62 ± 0.64
*C. granulosum* TP-CG10 (D)	3.3 ± 0.1	No data
*C. acnes* ATCC11828 (R)	6.0 ± 2.0	
*C. acnes* transconjugant	4.6 ± 0.6	0.64 ± 0.23
*C. granulosum* ATCC25564 (R)	4.3 ± 1.3	
*C. granulosum* transconjugant	5.6 ± 1.1	0.60 ± 0.29
*C. granulosum* TP-CG7 (D)	6.4 ± 1.7	1.38 ± 0.27
*C. acnes* ATCC11828 (R)	6.0 ± 2.0	
*C. acnes* transconjugant	4.1 ± 0.7^*b*^	0.59 ± 0.29^*c*^
*C. acnes* TP-CU426 (D)	8.3 ± 2.8	0.66 ± 0.25
*C. granulosum* ATCC25564 (R)	4.3 ± 1.3	
*C. granulosum* transconjugant	6.5 ± 1.7	0.51 ± 0.33
*C. granulosum* TP-CG108 (D)	5.8 ± 1.6	0.24 ± 0.07
*C. acnes* ATCC11828 (R)	6.0 ± 2.0	
*C. acnes* transconjugant	4.8 ± 0.5	0.58 ± 0.11^*c*^
*C. acnes* TP-CU814 (D)	3.8 ± 1.2	0.42 ± 0.07
*C. granulosum* ATCC25564 (R)	4.3 ± 1.3	
*C. granulosum* transconjugant	5.9 ± 0.5	0.64 ± 0.24

a D, donor; R, recipient.

b Significantly difference versus doubling time of *C. acnes* ATCC11828 using Welch’s *t* test (*P* < 0.05).

c Significantly difference versus copy number of pTZC1 of its donor strain using by Welch’s *t* test (*P* < 0.05).

The copy numbers of pTZC1 in each recipient strain and the transconjugant were compared (Table 4). Although some combinations showed significant increases or decreases in the copy number depending on the strain, no relationship between the copy number of pTZC1 and the MIC values for macrolides, clindamycin, and tetracyclines was observed.

## DISCUSSION

In 2008, the multidrug resistance plasmid pTZC1, which harbors *erm*(50) and *tet*(W), was found in the *C. acnes* strains isolated from acne patients in Osaka, Japan (16). Subsequently, *C. acnes* strains having pTZC1 were isolated in Tokyo and Hiroshima, and spread in Japan (12). In this study, the *C. acnes* and *C. granulosum* strains harboring pTZC1 were isolated together from two patients.

Therefore, pTZC1 was presumed to be transferred among different species. Of these two patients, one had use history of topical clindamycin and oral roxithromycin and the other had use history of oral minocycline (data not shown). Although antimicrobial use was not directly involved in the acquisition of antimicrobial resistance plasmid, increased selection pressure by antimicrobials may affect the advantage of survival in strains acquiring pTZC1.

pTZC1 sequences in *C. acnes* TP-CU426 and *C. granulosum* TP-CG7 isolated from the same patient showed 100% identity and had 500 bp deletion compared with pTZC1 in *C. acnes* TP-CU389 in our previous report (16). The 500-bp deletion sequence was located between the open reading frame (ORF) no. 10 and 11 in *C. acnes* TP-CU389. Therefore, the deleted sequence was to be a noncoding sequence that did not affect the plasmid function.

Therefore, pTZC1 was probably transferred between *C. acnes* TP-CU426 and *C. granulosum* TP-CG7 on the skin surface. In addition, the pTZC1 transfer test between *C. acnes* and *C. granulosum* showed bidirectional transconjugation. Transconjugants that acquired pTZC1 showed high-level resistance to clarithromycin and clindamycin (MICs ≥128 μg/mL). *erm*(50) encodes a 23S rRNA methylase and confers high-level resistance to macrolides and clindamycin (16). In contrast, *tet*(W) encodes a 16S rRNA protective protein, which is resistant to tetracyclines (16). *C. acnes* strains having *tet*(W) exhibited resistance or low susceptibility to tetracyclines, such as doxycycline and minocycline. Differences in tetracycline susceptibility may be related to *tet*(W) expression levels (4). Both the *C. acnes* TP-CU426 and *C. granulosum* TP-CG7 had not only acquisition of *tet*(W) but also *rpsJ* mutation (substitution of S10 protein), due to which these strains showed high MIC for doxycycline.

The acquisition of antimicrobial resistance factors may affect the growth ability. However, the *C. granulosum* TP-CG7 clinical isolate carrying pTZC1 exhibited similar growth levels to *C. granulosum* ATCC25564 as a type strain, suggesting that the possession of pTZC1 has little effect on growth ability. Therefore, strains with pTZC1 are likely capable of surviving and inhabiting the skin surface, similar to those without pTZC1.

The Japanese acne treatment guidelines recommend the use of topical clindamycin, oral macrolides, and oral tetracyclines (3). In addition to antimicrobial activity, these antimicrobials have anti-inflammatory activity and have been used to treat acne for a long time (19). The increased prevalence of strains carrying the multidrug-resistant plasmid pTZC1 may cause failure in antimicrobial treatment for acne vulgaris. Unlike *C. acnes*, *C. granulosum* is not known to develop during acne exacerbation. *C. granulosum* occupies a lower proportion of the normal skin flora and can contribute to skin health (20). In contrast, *C. granulosum*, like *C. acnes*, becomes an opportunistic pathogen and reportedly causes pathogenesis in surgical site infections of artificial joints and endocarditis (21 to 25). Although *Cutibacterium* species have recently gained attention as opportunistic pathogens, little is known about antimicrobial resistance in *C. granulosum* (26, 27). If *C. granulosum* causes opportunistic infections, an increase in the number of antimicrobial-resistant strains can hinder the selection of appropriate antimicrobials. Furthermore, pTZC1 transfer to *C. acnes* via *C. granulosum* may complicate the antimicrobial treatment of acne vulgaris.

In conclusion, our findings revealed that the multidrug resistance plasmid pTZC1 can be transferred between *C. acnes* and *C. granulosum* strains. Furthermore, because pTZC1 can be transferred between different species, it may aid in the prevalence of multidrug-resistant strains, and antimicrobial resistance genes may have been pooled on the skin surface. Further studies, such as *in vitro* skin models or *ex vivo* plasmid transfer studies, are necessary to confirm our findings.

## MATERIALS AND METHODS

### Strains and culture condition.

*Cutibacterium* strains were collected from 212 patients with acne who visited Toranomon Hospital between 2013 and 2018, and 264 patients who visited 13 dermatology clinics between 2016 and 2017 (4, 12, 28, 29). The colonies grown from specimens isolated from the patients under anaerobic conditions were determined using multiplex PCR for the identification of *Cutibacterium* species, according to our previous report (29). The strains could not be determined using multiplex PCR but were determined by BLAST homology analysis of 16S rRNA sequences. Single-locus sequence typing (SLST) of the *C. acnes* strains was performed using multiplex PCR according to the method described by Barnard et al. (30, 31).

Other species were determined by BLAST homology analysis of 16S rRNA gene sequences (31, 32). *C. acnes* ATCC 6919, ATCC 11828, and *C. granulosum* ATCC 25564 were used as the type strains for the quality control of antimicrobial susceptibility tests. All strains were incubated for 48 h at 35°C on modified GAM agar (Nissui Pharmaceutical, Tokyo, Japan) under anaerobic conditions in an anaerobic box (Hirasawa, Tokyo, Japan) and AnaeroPack-Anaero (Mitsubishi Gas Chemical Company, Inc., Tokyo, Japan).

For the plasmid conjugation test, *C. acnes* TP-CU389 (SLST; F1), TP-CU426 (F4), TP-CU814 (F1) strains, and *C. granulosum* TP-CG7, TP-CG9, and TP-CG108 were used as the donor strains, while rifampicin-resistant *C. acnes* ATCC11828 (K9) and rifampicin-resistant *C. granulosum* ATCC25564 were used as the recipient strains (14). These donor strains (TP-CU and TP-CG strains) were isolated from patients with acne vulgaris.

### Patients’ information.

A history of antimicrobial use within 6 months of occurrence of acne was obtained using a patient questionnaire. The questionnaire did not contain personally identifiable information, to protect patient privacy. The severity of acne was classified according to the establishment of grading criteria for acne severity (17).

### Antimicrobial susceptibility testing.

Antimicrobial susceptibility was evaluated by determining the MIC using the agar doubling dilution method according to the Clinical and Laboratory Standards Institute (CLSI) guidelines (33). Bacterial strains were resuspended in *Brucella* broth (Becton, Dickinson, Franklin Lakes, NJ, USA) containing 5 μg/mL hemin (Alfa Aesar, Haverhill, MA, USA), 1 μg/mL vitamin K1 (FUJIFILM Wako Pure Chemical, Osaka, Japan), and 5% lysed defibrinated horse blood (Nippon Bio-test Laboratories, Saitama, Japan). The resuspension solution was inoculated on *Brucella* agar (Becton, Dickinson) containing 5 μg/mL hemin, 1 μg/mL vitamin K1, and 5% lysed defibrinated sheep blood (Nippon Bio-test Laboratories). Minocycline hydrochloride (Fujifilm Wako Pure Chemical), clarithromycin, clindamycin hydrochloride monohydrate, and doxycycline hydrate (Tokyo Chemical Industries) were used as antimicrobial agents. Resistance breakpoints of *Cutibacterium* species were defined based on the *C. acnes* strains defined by the M100 of the Clinical and Laboratory Standard Institute (13, 33).

### Determination of antimicrobial resistance factors and pTZC1.

PCR was used to detect the macrolides-clindamycin resistance genes *erm*(X) and *erm*(50), tetracycline resistance gene *tet*(W), and *traE* of pTZC1 (16, 34). Furthermore, DNA sequencing was used to determine any 23S and 16S rRNA mutations associated with macrolide-clindamycin and tetracycline resistance, respectively (5, 13, 18).

### Whole-genome sequencing.

Genomic DNA was extracted as previously described (10). Whole-genome sequencing was performed at the Bioengineering Lab. Co. (Kanagawa, Japan) using GridION X5 (Oxford Nanopore Technologies, Oxford, UK) and DNBSEQ-G400 (MGI, Wuhan, China). The complete genome sequence was obtained by hybrid assembly of sequencing data with the Unicycler ver. v.0.4.7 default method. Contig graph was checked by Bandage (v.0.8.1). Completeness of genome assembled was confirmed by CheckM (v.1.1.2). ORFs annotation was analyzed using Prokka v.1.14.5. Default parameters were used for all software. The obtained plasmid sequences were compared to the known *C. acnes* TP-CU389 pTZC1 sequence (accession no. LC473083). A BLAST Ring Image Generator (BRIG; https://brig.sourceforge.net) was used for comparative analysis with known sequences.

### Transconjugation of *erm*(50) between *C. acnes* and *C. granulosum*.

The conjugation test for the multidrug resistant plasmid pTZC1, which codes *erm*(50), was performed by modifying the filter mating method previously described by Aoki et al. (14). Donor and recipient strains were incubated in a modified GAM medium (Nissui Pharmaceutical) for 48 h with shaking, and then inoculated at 1/100 of the volume into fresh medium. Bacterial cultures were incubated until an optical density at 600 nm (OD_600_) of 0.2 was obtained (OD_600_ was measured using Multiskan procured by Thermo Fisher Scientific). Donor and recipient strains were diluted 10-fold in GAM medium and collected at a rate of 4:1 in a bacterial population on a nitrocellulose membrane filter (diameter, 13 mm; pore size, 0.45 μm; Advantec, Tokyo, Japan). These filters were placed on modified GAM agar and incubated under anaerobic conditions for 3 days at 35°C. The culture on these filters was resuspended in fresh medium and spread over modified GAM agar containing 50 μg/mL rifampicin and 2 μg/mL clarithromycin. Transconjugants containing plasmids were defined as grown colonies that tested positive for *erm*(50), *tet*(W), and *tra*E by PCR (16). Transconjugation frequencies were presented as mean ± standard deviation (SD) averaged across three independent experiments.

### Growth ability of transconjugant.

To evaluate the influence of plasmid acquisition on bacterial growth, growth curves were plotted for recipient strains and transconjugants (5). After 48 h of anaerobic incubation with shaking, the bacterial culture was inoculated into a freshly modified GAM medium, and the OD_600_ of the cultures was measured over time. Doubling time was calculated as follows: [(*t2* – *t1*) × log_2_]/(logOD_600_ at *t2* -log OD_600_ at *t1*) (35). The data are presented as mean ± SD averaged across three independent experiments.

### The copy number analysis of pTZC1.

qPCR was performed to determine the copy number of pTZC1. Genomic DNA was extracted as previously described (15). The copy number was analyzed using real-time PCR and Thunderbird SYBR qPCR Mix (Toyobo, Osaka, Japan). The following specific primers were used: *tet*(W) for the pTZC1 marker, [tet(W)-rtF (5′-CCGACAGCAAAGTGGAAACAA), tet(W)-rtR (5′-GCAACTTGCGGATACTGACC)]; *oxc* for internal control of *C. acnes*, (oxc-rtF [5′-CTTGTCATCGGCGTATTCG], oxc-rtR [5′-CACTTCAAGCGGAAGGTGA]); and *recA* for internal control of *C. granulosum*, (recA-rtF [5′-GTAAGACCACGGTTGCCCT], and recA-rtR [5′-CAACACCCAACTTCTCCGCG]). The results are presented as the mean ± SD of three independent experiments.

### Statistical analysis.

Statistical comparisons between the two groups were performed using Welch’s *t* test and Fisher’s exact test in the js-STAR XR v.1.0.4j (http://www.kisnet.or.jp/nappa/software/star/) software.

### Data availability.

The genome sequences of the chromosomes and pTZC1 from *C. acnes* TP-CU426 were deposited in NCBI GenBank under accession numbers AP026712 and AP026713. The genome sequences of the chromosomes and pTZC1 from Cutibacterium granulosum were deposited in NCBI GenBank under accession numbers AP026710 and AP026711.

## References

[1] Zaenglein AL, Pathy AL, Schlosser BJ, Alikhan A, Baldwin HE, Berson DS, Bowe WP, Graber EM, Harper JC, Kang S, Keri JE, Leyden JJ, Reynolds RV, Silverberg NB, Stein Gold LF, Tollefson MM, Weiss JS, Dolan NC, Sagan AA, Stern M, Boyer KM, Bhushan R. 2016. Guidelines of care for the management of acne vulgaris. J Am Acad Dermatol 74:945–973.e33. doi:10.1016/j.jaad.2015.12.037. 26897386

[2] Schommer NN, Gallo RL. 2013. Structure and function of the human skin microbiome. Trends Microbiol 21:660–668. doi:10.1016/j.tim.2013.10.001. 24238601 PMC4744460

[3] Hayashi N, Akamatsu H, Iwatsuki K, Shimada-Omori R, Kaminaka C, Kurokawa I, Kono T, Kobayashi M, Tanioka M, Furukawa F, Furumura M, Yamasaki O, Yamasaki K, Yamamoto Y, Miyachi Y, Kawashima M. 2018. Japanese Dermatological Association guidelines: guidelines for the treatment of acne vulgaris 2017. J Dermatol 45:898–935. doi:10.1111/1346-8138.14355. 29782039

[4] Aoki S, Nakase K, Hayashi N, Nakaminami H, Noguchi N. 2021. Increased prevalence of doxycycline low-susceptible *Cutibacterium acnes* isolated from acne patients in Japan caused by antimicrobial use and diversification of tetracycline resistance factors. J Dermatol 48:1365–1371. doi:10.1111/1346-8138.15940. 33998707

[5] Nakase K, Sakuma Y, Nakaminami H, Noguchi N. 2016. Emergence of fluoroquinolone-resistant *Propionibacterium acnes* caused by amino acid substitutions of DNA gyrase but not DNA topoisomerase IV. Anaerobe 42:166–171. doi:10.1016/j.anaerobe.2016.10.012. 27793740

[6] Nakase K, Nakaminami H, Takenaka Y, Hayashi N, Kawashima M, Noguchi N. 2016. A novel 23S rRNA mutation in *Propionibacterium acnes* confers resistance to 14-membered macrolides. J Glob Antimicrob Resist 6:160–161. doi:10.1016/j.jgar.2016.05.005. 27530860

[7] Dreno B, Pecastaings S, Corvec S, Veraldi S, Khammari A, Roques C. 2018. *Cutibacterium acnes* (*Propionibacterium acnes*) and acne vulgaris: a brief look at the latest updates. J Eur Acad Dermatol Venereol 32(Suppl 2):5–14. doi:10.1111/jdv.15043. 29894579

[8] Dekio I, Okuda KI, Nishida M, Hamada-Tsutsumi S, Suzuki T, Kinoshita S, Tamura H, Ohnuma K, Murakami Y, Kinjo Y, Asahina A. 2021. Common features and intra-species variation of *Cutibacterium modestum* strains, and emended description of the species. Microorganisms 9:2343. doi:10.3390/microorganisms9112343. 34835467 PMC8620323

[9] Wei Q, Li Z, Gu Z, Liu X, Krutmann J, Wang J, Xia J. 2022. Shotgun metagenomic sequencing reveals skin microbial variability from different facial sites. Front Microbiol 13:933189. doi:10.3389/fmicb.2022.933189. 35966676 PMC9364038

[10] Koizumi J, Nakase K, Hayashi N, Nasu Y, Hirai Y, Nakaminami H. 2022. Multidrug-resistant *Cutibacterium avidum* isolated from patients with acne vulgaris and other infections. J Glob Antimicrob Resist 28:151–157. doi:10.1016/j.jgar.2021.12.021. 35017069

[11] Nakase K, Yoshida A, Saita H, Hayashi N, Nishijima S, Nakaminami H, Noguchi N. 2019. Relationship between quinolone use and resistance of *Staphylococcus epidermidis* in patients with acne vulgaris. J Dermatol 46:782–786. doi:10.1111/1346-8138.15000. 31254314

[12] Nakase K, Aoki S, Sei S, Fukumoto S, Horiuchi Y, Yasuda T, Tanioka M, Sugai J, Huh WW, Kakuta M, Nomoto M, Shimada T, Watanabe M, Kobayashi M, Murakami S, Takeo C, Tsubouchi R, Hayashi N, Noguchi N. 2020. Characterization of acne patients carrying clindamycin-resistant *Cutibacterium acnes*: a Japanese multicenter study. J Dermatol 47:863–869. doi:10.1111/1346-8138.15397. 32424832

[13] Nakase K, Nakaminami H, Noguchi N, Nishijima S, Sasatsu M. 2012. First report of high levels of clindamycin-resistant *Propionibacterium acnes* carrying *erm*(X) in Japanese patients with acne vulgaris. J Dermatol 39:794–796. doi:10.1111/j.1346-8138.2011.01423.x. 22142418

[14] Aoki S, Nakase K, Hayashi N, Noguchi N. 2019. Transconjugation of *erm*(X) conferring high-level resistance of clindamycin for *Cutibacterium acnes*. J Med Microbiol 68:26–30. doi:10.1099/jmm.0.000875. 30431414

[15] Koizumi J, Nakase K, Nakaminami H. 2022. Identification of a transferable linear plasmid carrying the macrolide-clindamycin resistance gene *erm*(X) in a *Cutibacterium acnes* isolate from a patient with acne vulgaris in Japan. Microbiol Resour Announc 11:e0009422. doi:10.1128/mra.00094-22. 35438511 PMC9119108

[16] Aoki S, Nakase K, Nakaminami H, Wajima T, Hayashi N, Noguchi N. 2020. Transferable multidrug-resistance plasmid carrying a novel macrolide-clindamycin resistance gene, *erm*(50), in *Cutibacterium acnes*. Antimicrob Agents Chemother 64:e01810-19. doi:10.1128/AAC.01810-19. 31844016 PMC7038278

[17] Hayashi N, Akamatsu H, Kawashima M, Acne Study Group. 2008. Establishment of grading criteria for acne severity. J Dermatol 35:255–260. doi:10.1111/j.1346-8138.2007.00403.x-i1. 18477223

[18] Nakase K, Nakaminami H, Takenaka Y, Hayashi N, Kawashima M, Noguchi N. 2017. *Propionibacterium acnes* is developing gradual increase in resistance to oral tetracyclines. J Med Microbiol 66:8–12. doi:10.1099/jmm.0.000392. 28218057

[19] Martins AM, Marto JM, Johnson JL, Graber EM. 2021. A review of systemic minocycline side effects and topical minocycline as a safer alternative for treating acne and rosacea. Antibiotics 10:757. doi:10.3390/antibiotics10070757. 34206485 PMC8300648

[20] Ogai K, Nana BC, Lloyd YM, Arios JP, Jiyarom B, Awanakam H, Esemu LF, Hori A, Matsuoka A, Nainu F, Megnekou R, Leke RGF, Ekali GL, Okamoto S, Kuraishi T. 2022. Skin microbiome profile of healthy Cameroonians and Japanese. Sci Rep 12:1364. doi:10.1038/s41598-022-05244-5. 35079063 PMC8789912

[21] Nystrom LM, Wyatt CM, Noiseux NO. 2013. Arthroplasty infection by *Priopionibacterium granulosum* treated with reimplantation despite ongoing purulent-appearing fluid collection. J Arthroplasty 28:198 e5–8. doi:10.1016/j.arth.2012.03.004. 22552222

[22] Broly M, Ruffier d'Epenoux L, Guillouzouic A, Le Gargasson G, Juvin ME, Leroy AG, Bemer P, Corvec S. 2020. *Propionibacterium*/*Cutibacterium* species-related positive samples, identification, clinical and resistance features: a 10-year survey in a French hospital. Eur J Clin Microbiol Infect Dis 39:1357–1364. doi:10.1007/s10096-020-03852-5. 32125556

[23] Chaudhry R, Dhawan B, Pandey A, Choudhary SK, Kumar AS. 2000. *Propionibacterium granulosum*: a rare cause of endocarditis. J Infect 41:284. doi:10.1053/jinf.2000.0728. 11120624

[24] Yedidya I, Goldberg E, Sharoni R, Sagie A, Vaturi M. 2015. Infective endocarditis caused by *Propionibacterium granulosum*. Isr Med Assoc J 17:642–643. 26665321

[25] Mutnal A, Patel P, Cardona L, Suarez J. 2011. Periprosthetic *Propionibacterium granulosum* joint infection after direct anterior total hip arthroplasty: a case report. JBJS Case Connect 1:e10. doi:10.2106/JBJS.CC.K.00029. 29252226

[26] Boman J, Nilson B, Sunnerhagen T, Rasmussen M. 2022. True infection or contamination in patients with positive *Cutibacterium* blood cultures—a retrospective cohort study. Eur J Clin Microbiol Infect Dis 41:1029–1037. doi:10.1007/s10096-022-04458-9. 35612767 PMC9250478

[27] Dekio I, Asahina A, Shah HN. 2021. Unravelling the eco-specificity and pathophysiological properties of *Cutibacterium* species in the light of recent taxonomic changes. Anaerobe 71:102411. doi:10.1016/j.anaerobe.2021.102411. 34265438

[28] Nakase K, Hayashi N, Akiyama Y, Aoki S, Noguchi N. 2017. Antimicrobial susceptibility and phylogenetic analysis of *Propionibacterium acnes* isolated from acne patients in Japan between 2013 and 2015. J Dermatol 44:1248–1254. doi:10.1111/1346-8138.13913. 28623856

[29] Koizumi J, Nakase K, Hayashi N, Nasu Y, Hirai Y, Nakaminami H. 2023. Prevalence of antimicrobial-resistant *Cutibacterium* isolates and development of multiplex PCR method for *Cutibacterium* species identification. J Infect Chemother 29:198–204. doi:10.1016/j.jiac.2022.10.018. 36336238

[30] Scholz CF, Jensen A, Lomholt HB, Bruggemann H, Kilian M. 2014. A novel high-resolution single locus sequence typing scheme for mixed populations of *Propionibacterium acnes in vivo*. PLoS One 9:e104199. doi:10.1371/journal.pone.0104199. 25111794 PMC4128656

[31] Barnard E, Nagy I, Hunyadkurti J, Patrick S, McDowell A. 2015. Multiplex touchdown PCR for rapid typing of the opportunistic pathogen Propionibacterium acnes. J Clin Microbiol 53:1149–1155. doi:10.1128/JCM.02460-14. 25631794 PMC4365214

[32] Noguchi N, Nakaminami H, Nakase K, Sasatsu M. 2011. Characterization of *Enterococcus* strains contained in probiotic products. Biol Pharm Bull 34:1469–1473. doi:10.1248/bpb.34.1469. 21881235

[33] Clinical and Laboratory Standards Institute. 2021. M100—performance standard for antimicrobial susceptibility testing, 31th ed. CLSI, Malvern, PA.

[34] Ishida N, Nakaminami H, Noguchi N, Kurokawa I, Nishijima S, Sasatsu M. 2008. Antimicrobial susceptibilities of *Propionibacterium acnes* isolated from patients with acne vulgaris. Microbiol Immunol 52:621–624. doi:10.1111/j.1348-0421.2008.00081.x. 19120976

[35] Matsuo M, Hishinuma T, Katayama Y, Cui L, Kapi M, Hiramatsu K. 2011. Mutation of RNA polymerase beta subunit (rpoB) promotes hVISA-to-VISA phenotypic conversion of strain Mu3. Antimicrob Agents Chemother 55:4188–4195. doi:10.1128/AAC.00398-11. 21746940 PMC3165293

